# Incidence- and In-hospital Mortality-Related Risk Factors of Acute Kidney Injury Requiring Continuous Renal Replacement Therapy in Patients Undergoing Surgery for Acute Type a Aortic Dissection

**DOI:** 10.3389/fcvm.2021.749592

**Published:** 2021-11-23

**Authors:** Xuelian Chen, Jiaojiao Zhou, Miao Fang, Jia Yang, Xin Wang, Siwen Wang, Linji Li, Tao Zhu, Ling Ji, Lichuan Yang

**Affiliations:** ^1^Division of Nephrology, Department of Medicine, West China Hospital of Sichuan University, Chengdu, China; ^2^Division of Ultrasound, West China Hospital, Sichuan University, Chengdu, China; ^3^Department of Orthopedics, Second People's Hospital of Chengdu, Chengdu, China; ^4^Department of Pediatric Nephrology, West China Second University Hospital, Sichuan University, Chengdu, China; ^5^Department of Anesthesiology, West China Hospital, Sichuan University, The Research Units of West China (2018RU012), Chinese Academy of Medical Sciences, Chengdu, China; ^6^Division of Nephrology, West China Hospital of Sichuan University, Chengdu, China

**Keywords:** acute type A aortic dissection, surgery, acute kidney injury, continuous renal replacement therapy, CRRT, risk factors

## Abstract

**Background:** Few studies on the risk factors for postoperative continuous renal replacement therapy (CRRT) in a homogeneous population of patients with acute type A aortic dissection (AAAD). This retrospective analysis aimed to investigate the risk factors for CRRT and in-hospital mortality in the patients undergoing AAAD surgery and to discuss the perioperative comorbidities and short-term outcomes.

**Methods:** The study collected electronic medical records and laboratory data from 432 patients undergoing surgery for AAAD between March 2009 and June 2021. All the patients were divided into CRRT and non-CRRT groups; those in the CRRT group were divided into the survivor and non-survivor groups. The univariable and multivariable analyses were used to identify the independent risk factors for CRRT and in-hospital mortality.

**Results:** The proportion of requiring CRRT and in-hospital mortality in the patients with CRRT was 14.6 and 46.0%, respectively. Baseline serum creatinine (SCr) [odds ratio (*OR*), 1.006], cystatin C (*OR*, 1.438), lung infection (*OR*, 2.292), second thoracotomy (*OR*, 5.185), diabetes mellitus (*OR*, 6.868), AKI stage 2–3 (*OR*, 22.901) were the independent risk factors for receiving CRRT. In-hospital mortality in the CRRT group (46%) was 4.6 times higher than in the non-CRRT group (10%). In the non-survivor (*n* = 29) and survivor (*n* = 34) groups, New York Heart Association (NYHA) class III-IV (*OR*, 10.272, *P* = 0.019), lactic acidosis (*OR*, 10.224, *P* = 0.019) were the independent risk factors for in-hospital mortality in patients receiving CRRT.

**Conclusion:** There was a high rate of CRRT requirement and high in-hospital mortality after AAAD surgery. The risk factors for CRRT and in-hospital mortality in the patients undergoing AAAD surgery were determined to help identify the high-risk patients and make appropriate clinical decisions. Further randomized controlled studies are urgently needed to establish the risk factors for CRRT and in-hospital mortality.

## Introduction

Acute kidney injury (AKI) is one of the most common postoperative complications following cardiac surgery and is associated with increased morbidity and mortality (1). There is currently no consensus on the definition of cardiac surgery-associated AKI (CSA-AKI). The Kidney Disease: Improving Global Outcomes criteria (KDIGO) represent the current epidemiological and clinical standard for diagnosing AKI, including CSA-AKI (2, 3). Acute type A aortic dissection (AAAD) is the most dramatic emergency in cardiac surgery due to the high in-hospital mortality rate of ~26% (4). Compared with other heart surgeries, the risk of AKI is higher after aortic dissection surgery (5, 6). Between 1999 and 2008, the incidence of AKI and AKI requiring dialysis (AKI-D) after cardiac surgery has increased from 30 and 5% to 47 and 14%, respectively (7). Continuous renal replacement therapy (CRRT) is widely used for hemodynamically unstable patients with considerable fluid accumulation (3, 8). However, the mortality of receiving CRRT after AKI is 40–70% (9), and second, the high cost of CRRT and the limited medical resources associated with CRRT impose a heavy social burden (10). Early identification of critically ill patients of high risk for CRRT and in-hospital mortality after cardiac surgery can be beneficial in improving the overall prognosis.

Few studies on the risk factors for postoperative CRRT in a homogeneous population of patients with AAAD. This retrospective analysis was designed to describe the incidence of AKI requiring CRRT in the patients undergoing AAAD surgery, evaluate the demographic and perioperative factors associated with the patients with AKI requiring CRRT, identify the association of CRRT with a duration of mechanical ventilation, in-hospital mortality, and 1-year readmission, and analyze the risk factors for CRRT and in-hospital mortality.

## Materials and Methods

### Study Population

**Figure 1:**
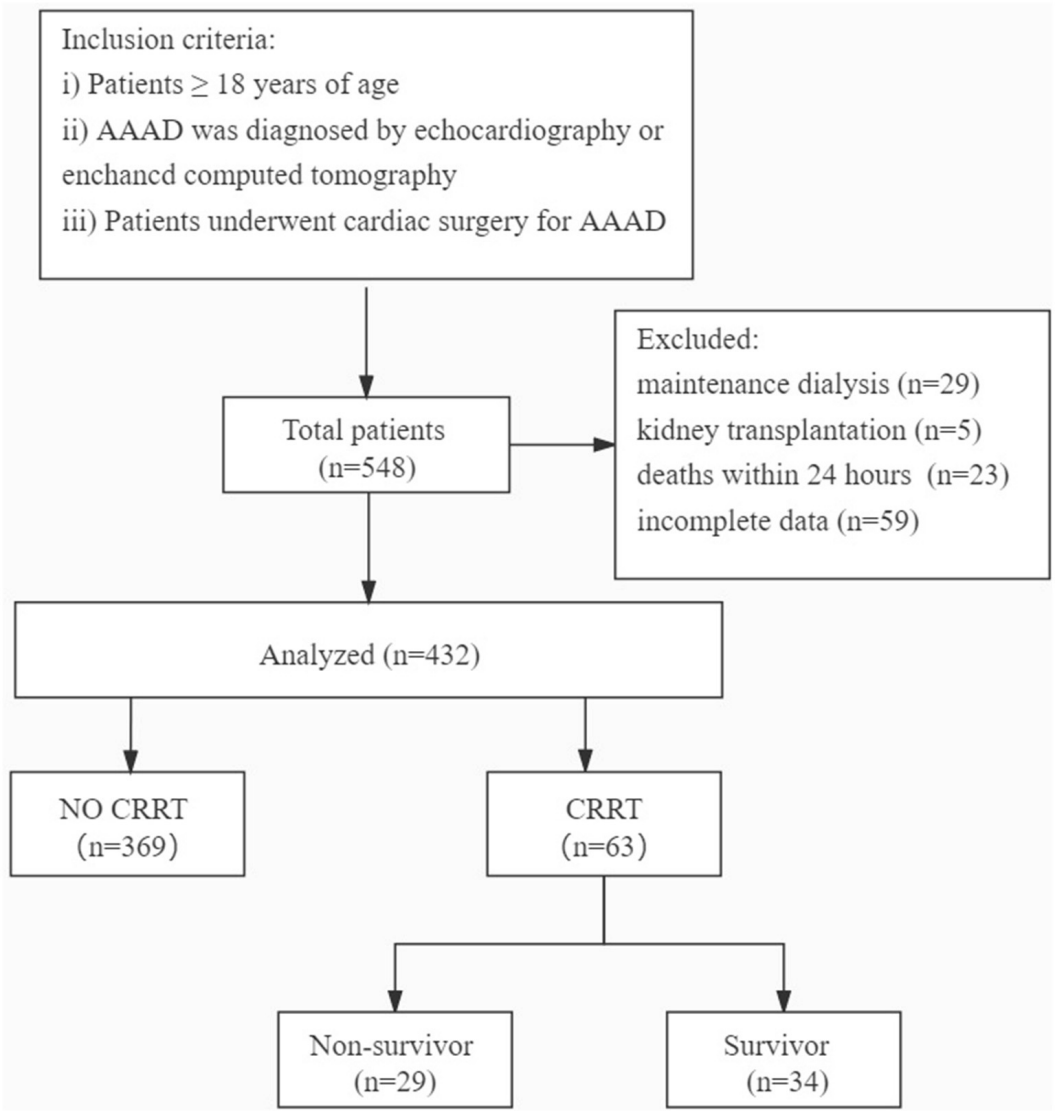
Flow diagram of the study.

For this study, in Figure 1, we retrospectively collected the electronic medical records and laboratory results of 432 patients aged ≥18 years who underwent cardiac surgery for AAAD diagnosed by echocardiography or enhanced CT at the West China Hospital of Sichuan University (Sichuan, China) between March 2009 and June 2021. The exclusion criteria were as follows: (i) the patients who had received maintenance dialysis within the last month or were on dialysis before surgery (*n* = 29); (ii) the patients who had received kidney transplantation (*n* = 5); (iii) the patients who died within 24 h of admission to the hospital (*n* = 23); and (iv) the patients with incomplete data (*n* = 59). The present study was approved by the Ethics Committees of the West China Hospital of Sichuan University. Sichuan, China.

### Data Collection

The data of the patients included in the present study, including the patient characteristics, perioperative factors, laboratory data, postoperative complications or comorbidities, and outcomes, were extracted from the electronic medical records system of our institution. The laboratory data of the CRRT group were obtained prior to the occurrence of AKI.

### Measurements and Variable Definitions

Acute kidney injury was defined according to the KDIGO criteria (2) as follows: a serum creatinine (SCr) levels increase of ≥0.3 mg/dl within 48 h, or ≥1.5-fold the baseline level, which is known or hypothesized to have occurred within the prior 7 days, or urine output <0.5 ml/kg/h for 6 consecutive h. AKI severity was staged according to the following criteria (2) (Table 1).

**Table 1:** Kidney Disease: Improving Global Outcomes (KDIGO) stages of acute kidney injury (AKI) according to serum creatinine (SCr) levels and urine output.

**Stage**	**Serum creatinine**	**Urine output**
1	1.5–1.9 times baseline	<0.5 ml/kg/h for 6–12 h
	Or ≥0.3 mg/dl (≥26.5 mmol/l) increase	
2	2.0–2.9 times baseline	<0.5 ml/kg/h for ≥12 h
3	3.0 times baseline	<0.3 ml/kg/h for ≥24 hOr anuria for ≥12 h
	Or increase in serum creatinine to ≥4.0 mg/dl (≥353.6 mmol/l)	
	Or initiation of renal replacement therapy or in patients <18 years, decrease in eGFR to <35 ml/min per 1.73 m^2^	

*KDIGO, Kidney Disease: Improving Global Outcomes; eGFR, estimated glomerular filtration rate*.

In this retrospective analysis, the urine output criteria for defining AKI were not used, as 6- and 12-h urine outputs were not reliably recorded, and certain patients were treated with diuretics and CRRT. The decision for CRRT is determined by the assessment of the severity of AKI by the clinician and the comprehensive condition of patients. As some patients with AAAD were referred to our hospital from other hospitals, the lowest SCr level in the 2 days prior to admission was used as the baseline level if data were available; if not, we considered the first SCr available at admission as the baseline value. Drinking is defined as having consumed alcohol at least one time a week in the last year. CKD was defined by past medical history. Preoperative liver insufficiency was defined as a preoperative elevation of >80 U/L of aspartate aminotransferase (AST) or (and) alanine aminotransferase (ALT) in the patients. Deep hypothermic circulatory arrest (DHCA) is a measure to protect the brain by arresting extracorporeal circulation after cooling the brain temperature to 14.1–20°C in aortic arch replacement, for brain preservation. The diagnosis of postoperative acute myocardial infarction (AMI) was based on a history of typical chest pain, diagnostic ECG changes, serum cardiac biomarkers, and abnormalities on echocardiography and coronary angiography. Cerebral ischemia or hemorrhage resulting in complete or incomplete loss of brain function was defined as an acute cerebrovascular accident (CVA).

The patients were divided into the CRRT and non-CRRT groups according to whether they required CRRT or not; the patients in the CRRT group were divided into the survivor and non-survivor groups. The independent risk factors and perioperative complications and short-term outcomes were analyzed.

The primary outcomes were the occurrence of severe AKI, requiring CRRT, and in-hospital mortality. The secondary outcomes were the duration of mechanical ventilation, hospitalization costs, and 1-year re-admission.

### Statistical Analysis

The baseline demographic and clinical data of patients were presented as numbers and percentages for categorical variables. The categorical variables were compared using chi-square tests. Data distribution normality was verified using the Kolmogorov–Smirnov test. The normally distributed continuous variables were presented as mean ± SD and the non-normally distributed continuous variables were represented as the median and interquartile range (IQR) and compared accordingly using independent samples *t*-test or Mann–Whitney *U*-test. A stepwise forward binary logistic regression analysis was applied to analyze the risk factors for patients receiving postoperative CRRT and in-hospital mortality. The omnibus tests of model coefficients were used to evaluate the multivariable binary logistic regression model, with P <0.05 indicating that the binary logistic regression model was generally significant. Hosmer and Lemeshow test and −2 log likelihood (−2LL) provided an evaluation of the goodness of fit of the logistic regression model, and *P* > 0.05 would indicate a good fit. Only those variables found to be statistically significant in the univariable analysis and with a variance inflation factor (VIF) <10 in the linear regression analysis were used in logistic regression analysis.

Statistical significance was set at *P* ≤ 0.05. Statistical analysis was performed by the SPSS software package, version 26.0 (IBM Corp., Chicago, IL, USA) and GraphPad Prism 8.0 software (GraphPad Software, Inc., San Diego, CA, USA).

## Results

### Demographic and Clinical Characteristics

A total of 432 patients with AAAD were included in the final sample. The demographic and clinical characteristics and laboratory data of patients hospitalized with AAAD are summarized in Table 2.

**Table 2:** Demographics, preoperative factors, and laboratory data of 432 patients.

	**ALL (*n* = 432)**	**No CRRT (*n* = 369)**	**CRRT (*n* = 63)**	***P* value**
Age, year, mean ± SD	48.19 ± 10.97	47.69 ± 10.59	51.10 ± 12.63	**0.023**
Gender, n (%)				**0.036**
Male	341 (78.9%)	285 (77.2%)	56 (88.9%)	
Female	91 (21.1%)	84 (22.8%)	7 (11.1%)	
BMI, kg/m^2^, mean ± SD	24.85 ± 3.78	24.84 ± 3.65	24.89 ± 4.53	0.916
Smoking, n (%)	210 (48.6%)	181 (49.1%)	29 (46.0%)	0.658
Drinking, n (%)	136 (31.5%)	122 (33.1%)	14 (22.2%)	0.087
**Medical history, n (%)**
Hypertension	250 (57.9%)	209 (56.6%)	41 (65.1%)	0.210
Poor blood pressure control	92 (21.3%)	72 (19.5%)	20 (31.7%)	**0.028**
A left ventricular ejection fraction of <35%	28 (6.5%)	22 (6.0%)	6 (9.5%)	0.289
Marfan syndrome	18 (4.2%)	14 (3.8%)	4 (6.3%)	0.348
Previous cardiac surgery	27 (6.3%)	23 (6.2%)	4 (6.3%)	0.972
Aortic regurgitation	176 (40.7%)	150 (40.7%)	26 (41.3%)	0.926
CKD	30 (6.9%)	22 (6.0%)	8 (12.7%)	0.052
COPD	28 (6.5%)	21 (5.7%)	7 (11.1%)	0.106
Diabetes mellitus	20 (4.6%)	11 (3.0%)	9 (14.3%)	**0.000**
Chronic liver disease	22 (5.1%)	18 (4.9%)	4 (6.3%)	0.624
**Preoperative factors**
Hemorrhagic shock, n (%)	7 (1.6%)	4 (1.1%)	3 (4.8%)	0.094
Pericardial tamponade, n (%)	20 (4.6%)	17 (4.6%)	3 (4.8%)	0.957
NYHA class III-IV, n (%)	111 (25.7%)	85 (23.0%)	26 (41.3%)	**0.002**
Liver insufficiency, n (%)	51 (11.8%)	36 (9.8%)	15 (23.8%)	**0.001**
**Renal artery involvement, n (%)**	184 (42.6%)	154 (41.7%)	30 (47.6%)	0.383
Renal malperfusion, n (%)	103 (23.8%)	83 (22.5%)	20 (31.7%)	0.113
No renal malperfusion, n (%)	81 (18.8%)	71 (19.2%)	10 (15.9%)	0.527
**Lab data (mean** **±** **SD) or median (IQR)**
Baseline SCr, μmol/L	82 (65, 104)	79 (64, 99)	97 (83, 131)	**0.000**
BUN, mmol/L	6.32 (4.95, 8.60)	6.15 (4.90, 8.30)	7.50 (5.76, 11.41)	**0.000**
UA, umol/L	353 (280, 442)	349 (276, 430)	405 (301, 480)	**0.047**
Cys-C, mg/L	1.30 (0.92, 2.08)	1.2 (0.90, 1.73)	3.11 (1.54, 4.22)	**0.000**
ALB, g/L	37.90 (34.23, 41.10)	38.10 (34.35, 41.15)	36.10 (30.90, 40.30)	0.056
Proteinuria, n (%)	178 (41.2%)	142 (38.5%)	36 (57.1%)	**0.005**
Hematuria, n (%)	123 (28.5%)	104 (28.2%)	19 (30.2%)	0.748

*The continuous variables that follow a normal distribution include age, and BMI which were expressed as mean ± SD. The continuous variables that do not follow a normal distribution were represented as median and IQR. The categorical variables were presented as numbers and percentages. Liver insufficiency was defined as a preoperative elevation of >80 U/L of aspartate aminotransferase (AST) or (and) alanine aminotransferase (ALT) in patients*.

*AKI, acute kidney injury; Scr, serum creatinine; SD, standard deviations; BMI, body mass index; CKD, chronic kidney disease; COPD, chronic obstructive pulmonary disease; NYHA, New York Heart Association; AST, aspartate aminotransferase; ALT, alanine aminotransferase; DHCA, Deep hypothermic circulatory arrest; CPB, cardiopulmonary bypass; ALB, albumin; BUN, blood urea nitrogen; UA, uric acid; Cys-C, cystatin C; CRRT, continuous renal replacement therapy. Bold values represented P <0.05*.

The mean age of all patients included in the study was 48.19 ± 10.97 years. The median body mass index (BMI) was 24.85 ± 3.78 kg/m^2^. There were 250 (57.9%) patients with hypertension and 92 (21.3%) with poor blood pressure control. A small number of patients had chronic kidney injury (CKD, 6.9%), chronic obstructive pulmonary disease (COPD, 6.5%), diabetes (4.6%), chronic liver disease (5.1%), and other comorbidities. The median of baseline SCr level was 82 (65, 104) μmol/L. The median of cystatin C (Cys-C) level was 1.30 (0.92, 2.08) mg/L. According to aortic computed tomography angiography (CTA) findings, 184 patients were diagnosed with renal artery involvement, 103 of which had coexisting renal malperfusion.

### Primary and Second Outcomes of Patients With AAAD

Among the recorded patients, 47.9% developed postoperative severe AKI (AKI stages 2–3). The total number of in-hospital mortality and requiring for CRRT was 66 (15.3%) and 63 (14.6%), respectively. In-hospital mortality in the CRRT group (46%) was 4.6 times higher than in the non-CRRT group (10%). Compared with the non-CRRT group, the CRRT group had a longer duration of mechanical ventilation and higher hospitalization costs. No statistical difference was observed in the 1-year readmission rate between the two groups (*P* = 0.762) (Table 3).

**Table 3:** Interoperative factors, postoperative comorbidities, and outcomes of 432 patients.

	**ALL (*n* = 432)**	**Non-CRRT (*n* = 369)**	**CRRT (*n* = 63)**	***P* value**
**Interoperative factors**
Total aortic arch replacement n (%)	319 (73.8%)	270 (73.2%)	49 (77.8%)	0.442
DHCA n (%)	105 (24.3%)	93 (25.2%)	12 (19.0%)	0.292
CPB duration ≥180 min n (%)	387 (89.6%)	336 (91.1%)	51 (81.0%)	**0.015**
RBC transfusion (units)	3.0 (1.0, 4.5)	3.0 (0.0, 4.0)	3.5 (2.0, 5.5)	0.150
**Postoperative comorbidities, n (%)**
MAP within 2 h after surgery ≤ 65 mmHg	94 (21.8%)	67 (18.2%)	27 (42.9%)	**0.000**
Second thoracotomy	22 (5.1%)	11 (3.0%)	11 (17.5%)	**0.000**
AMI	14 (3.2%)	8 (2.2%)	6 (9.5%)	**0.002**
CVA	39 (9.0%)	32 (8.7%)	7 (11.1%)	0.532
Lung infection	132 (30.6%)	94 (25.5%)	38 (60.3%)	**0.000**
Lactic acidosis	104 (24.1%)	79 (21.4%)	25 (39.7%)	**0.002**
ARDS	65 (15.0%)	50 (13.6%)	15 (23.8%)	**0.035**
Hepatic failure	40 (9.3%)	29 (7.9%)	11 (17.5%)	**0.015**
AKI stage 2–3	207 (47.9%)	146 (39.6%)	61 (96.8%)	**0.000**
**Outcomes**
Duration of mechanical ventilation, days	4 (2, 6)	3 (2, 5)	7 (5, 14)	**0.000**
Hospitalization costs, $	30410.21 (24734.17, 39734.99)	29635.79 (24052.40, 36689.51)	41147.65 (33731.50, 49527.07)	**0.000**
1-year re-admission n (%)	53 (12.3%)	46 (12.5%)	7 (11.1%)	0.762
In-hospital mortality n (%)	66 (15.3%)	37 (10.0%)	29 (46.0%)	**0.000**

*The continuous variables and categorical variables were presented as mean ± SD, median and IQR, numbers and percentages, respectively. AKI, acute kidney injury; Scr, serum creatinine; SD, standard deviations; DHCA, deep hypothermic circulatory arrest; CPB, cardiopulmonary bypass; MAP, mean arterial pressure; AMI, acute myocardial infarction; CVA, acute cerebrovascular accident; ARDS, acute respiratory distress syndrome; CRRT, continuous renal replacement therapy. Bold values represented P <0.05*.

^$^*Represents United States Dollar (USD)*.

### Risk Factors for Postoperative AKI With CRRT in Patients With AAAD

The multivariable analysis revealed baseline SCr [odds ratio (*OR*), 1.006; *P* = 0.011], Cys-C (*OR*, 1.438; *P* = 0.001), lung infection (*OR*, 2.292; *P* = 0.017), second thoracotomy (*OR*, 5.185; *P* = 0.004), diabetes mellitus (*OR*, 6.868; *P* = 0.001), AKI stage 2–3 (*OR*, 22.901; *P* = 0.000) were the independent risk factors for receiving CRRT (Table 4).

**Table 4:** A multivariable analysis of risk factors associated with continuous renal replacement therapy (CRRT).

**Variables**	**β**	**Odds ratio**	**95% confidence interval**		***P* value**
			**Lower**	**Upper**	
Baseline SCr	0.006	1.006	1.001	1.011	**0.011**
Cys-C	0.364	1.438	1.167	1.774	**0.001**
Lung infection	0.829	2.292	1.157	4.539	**0.017**
Second thoracotomy	1.646	5.185	1.700	15.814	**0.004**
Diabetes mellitus	1.927	6.868	2.182	21.621	**0.001**
AKI stage 2–3	3.131	22.901	5.220	100.466	**0.000**

*Age, gender, poor blood pressure control, diabetes mellitus, NYHA class III–IV, liver insufficiency, baseline SCr, Cys-C, BUN, UA, proteinuria, MAP within 2 h after surgery ≤ 65 mmHg, second thoracotomy, AMI, lung infection, lactic acidosis, ARDS, hepatic failure, AKI stage 2–3 were included to establish a stepwise forward binary logistic regression (Omnibus tests of model coefficients: X^2^ = 137.693, P = 0.000; Hosmer–Lemeshow goodness-of-fit test: X^2^ = 6.711, P = 0.568; −2 log likelihood = 221.224). In the linear regression analysis of these variables, the variance inflation factor (VIF) was <10. Therefore, there were no problems of multicollinearity among these independent variables. The odds ratio (OR) and 95% CI were measured through binary logistic regression. The OR of the continuous variable represented that for a single unit increase, the probability of a positive event will increase by approximately OR-1 times*.

*NYHA, New York Heart Association; PLT, platelet; ALB, albumin; SCr, serum creatinine; Cys-C, cystatin C; CPB, cardiopulmonary bypass; MAP, mean arterial pressure; AMI, acute myocardial infarction; ARDS, acute respiratory distress syndrome; AKI, acute kidney injury. Bold values represented P <0.05*.

### Risk Factors for In-hospital Mortality in Patients With CRRT After Surgery for AAAD

In this study, 63 patients with CRRT were divided into the survivor (34/63, 54.0%) and non-survivor groups (29/63, 46.0%). The characteristics, perioperative factors, laboratory data, and outcomes of the patients with CRRT are shown in Table 5. In the non-survivors and survivors groups, there were statistically significant differences in the history of drinking (41.4 vs. 5.9%), preoperative NYHA class III-IV (75.9 vs. 11.8%), cardiopulmonary bypass (CPB) duration ≥ 180 min (93.1 vs. 70.6%), duration of mechanical ventilation (16.5 vs. 10 days), MAP ≤ 65 mmHg within 2 h postoperatively (62.1 vs. 26.5%), hepatic failure (34.5 vs. 2.9%), acute respiratory distress syndrome (ARDS) (37.9 vs. 11.8%), lactic acidosis (62.1 vs. 20.6%), and CVA (20.7 vs. 2.9%).

**Table 5:** Demographics, perioperative factors, and outcomes of 63 patients with CRRT.

	**ALL (*n* = 63)**	**Non-survivor (*n* = 29)**	**Survivor (*n* = 34)**	***P* value**
Age, year, mean ± SD	51.10 ± 12.63	53.90 ± 12.48	48.71 ± 12.45	0.104
Gender, n (%)				0.326
Male	56 (88.9%)	27 (93.1%)	29 (85.3%)	
Female	7 (11.1%)	2 (6.9%)	5 (14.7%)	
BMI, kg/m^2^, median (IQR)	25.06 (22.49, 27.17)	25.06 (22.49, 27.78)	24.86 (22.91, 26.45)	0.610
Smoking, n (%)	29 (46%)	14 (48.3%)	15 (44.1%)	0.741
Drinking	14 (22.2%)	12 (41.4%)	2 (5.9%)	**0.001**
**Medical history, n (%)**				
Hypertension	41 (65.1%)	19 (65.5%)	22 (64.7%)	0.946
Poor blood pressure control	20 (31.7%)	11 (37.9%)	9 (26.5%)	0.330
A left ventricular ejection fraction of <35%	6 (9.5%)	5 (17.2%)	1 (2.9%)	0.054
Marfan syndrome	4 (6.3%)	1 (3.4%)	3 (8.8%)	0.383
Previous cardiac surgery	4 (6.3%)	3 (10.3%)	1 (2.9%)	0.230
Aortic regurgitation	26 (41.3%)	8 (27.6%)	18 (52.9%)	**0.042**
CKD	8 (12.7%)	2 (6.9%)	6 (17.6%)	0.201
COPD	7 (11.1%)	4 (13.8%)	3 (8.8%)	0.532
Diabetes mellitus	9 (14.3%)	6 (20.7%)	3 (8.8%)	0.180
Chronic liver disease	4 (6.3%)	2 (6.9%)	2 (5.9%)	0.869
**Preoperative factors**				
Hemorrhagic shock, n (%)	3 (4.8%)	1 (3.4%)	2 (5.9%)	1.000
Pericardial tamponade, n (%)	3 (4.8%)	2 (6.9%)	1 (2.9%)	0.590
NYHA class III-IV n (%)	26 (41.3%)	22 (75.9%)	4 (11.8%)	**0.000**
Liver insufficiency, n (%)	15 (23.8%)	10 (34.5%)	5 (14.7%)	0.066
**Renal artery involvement, n (%)**	30 (42.7%)	13 (44.8%)	17 (50.0%)	0.682
Renal malperfusion, n (%)	20 (31.7%)	7 (24.1%)	13 (38.2%)	0.231
No renal malperfusion, n (%)	10 (15.9%)	6 (20.7%)	4 (11.8%)	0.492
**Lab data (mean** **±** **SD) or median (IQR)**				
Baseline Scr, μmol/L	97 (83, 131)	97 (86, 111)	98 (72, 160)	0.720
BUN, mmol/L	7.5 (5.76, 11.41)	8.09 (5.92, 10.75)	7.40 (5.58, 11.70)	0.901
UA, umol/L	390.54 ± 129.90	380.25 ± 125.79	399.31 ± 134.55	0.566
Cys-C, mg/L	3.11 (1.54, 4.22)	2.95 (1.87, 4.15)	3.28 (1.09, 4.33)	0.940
ALB, g/L	36.10 (30.90, 40.30)	36.10 (30.70, 40.80)	36.10 (30.67, 40.23)	0.730
Proteinuria, n (%)	36 (57.1%)	14 (48.3%)	22 (64.7%)	0.189
Hematuria, n (%)	19 (30.2%)	9 (31.0%)	10 (29.4%)	0.889
**Interoperative factors**				
Total aortic arch replacement n (%)	49 (77.8%)	21 (72.4%)	28 (82.4%)	0.344
DHCA n (%)	12 (19.0%)	6 (20.7%)	6 (17.6%)	0.759
CPB duration ≥180 min n (%)	51 (81.0%)	27 (93.1%)	24 (70.6%)	**0.023**
RBC transfusion (units)	3.5 (3.0, 5.5)	3.0 (1.8, 5.5)	3.8 (2.0, 5.6)	0.527
**Postoperative comorbidities, n (%)**				
MAP within 2 h after surgery ≤ 65 mmHg	27 (42.9%)	18 (62.1%)	9 (26.5%)	**0.004**
Second thoracotomy	11 (17.5%)	5 (17.2%)	6 (17.6%)	0.966
AMI	6 (9.5%)	4 (13.8%)	2 (5.9%)	0.286
Lung infection	38 (60.3%)	21 (72.4%)	17 (50.0%)	0.070
Hepatic failure	11 (17.5%)	10 (34.5%)	1 (2.9%)	**0.001**
ARDS	15 (23.8%)	11 (37.9%)	4 (11.8%)	**0.015**
Lactic acidosis	25 (39.7%)	18 (62.1%)	7 (20.6%)	**0.001**
CVA	7 (11.1%)	6 (20.7%)	1 (2.9%)	**0.042**
**Outcomes**				
Hospitalization costs, $	41276.87 ± 14961.75	42604.69 ± 15729.64	40144.32 ± 14414.22	0.520
Duration of mechanical ventilation, days	7 (5, 14)	16.5 (9, 21)	10 (6, 20)	**0.049**

*The continuous variables that follow a normal distribution include age, uric acid (UA), hospitalization costs were expressed as mean ± SD. The continuous variables that do not follow a normal distribution were represented as median and IQR. The categorical variables were presented as numbers and percentages. Liver insufficiency was defined as a preoperative elevation of >80 U/L of AST or (and) ALT in the patients*.

*AKI, acute kidney injury; Scr, serum creatinine; SD, standard deviations; BMI, body mass index; CKD, chronic kidney disease; COPD, chronic obstructive pulmonary disease; NYHA, New York Heart Association; AST, aspartate aminotransferase; ALT, alanine aminotransferase; DHCA, Deep hypothermic circulatory arrest; CPB, cardiopulmonary bypass; ALB, albumin; BUN, blood urea nitrogen; UA, uric acid; Cys-C, cystatin C; CRRT, continuous renal replacement therapy; DHCA, Deep hypothermic circulatory arrest; CPB, cardiopulmonary bypass; MAP, mean arterial pressure; AMI, acute myocardial infarction; CVA, acute cerebrovascular accident; ARDS, acute respiratory distress syndrome. Bold values represented P <0.05. ^$^Represents United States Dollar (USD)*.

In Table 6, the multivariable analysis showed that the independent risk factors for in-hospital mortality in the patients with CRRT included preoperative NYHA class III-IV (*OR*, 10.272; *P* = 0.019), lactic acidosis (*OR*, 10.224; *P* = 0.019). The patients with two or more predisposing factors including the history of drinking, CPB duration ≥ 180 min, MAP ≤ 65 mmHg within 2 h postoperatively, ARDS, hepatic failure, and acute cerebrovascular accident (CVA), were high-risk patients for in-hospital death in CRRT (*OR*, 19.816; *P* = 0.002).

**Table 6:** The multivariable analysis of risk factors associated with in-hospital mortality in the patients with CRRT.

**Variables**	**β**	**Odds ratio**	**95% confidence interval**		***P* value**
			**Lower**	**Upper**	
Lactic acidosis	2.325	10.224	1.459	71.641	**0.019**
NYHA class III-IV	2.329	10.272	1.469	71.802	**0.019**
Risk factors ≥ 2 including Drinking CPB duration ≥180 min MAP within 2 h after surgery ≤ 65 mmHg Hepatic failure ARDS CVA	2.986	19.816	3.035	129.389	**0.002**

*Drinking history, NYHA class III–IV, CPB duration ≥180 min, duration of mechanical ventilation, MAP within 2 h after surgery ≤ 65 mmHg, CVA, lactic acidosis, ARDS, and hepatic failure were included to establish a stepwise forward binary logistic regression (Omnibus tests of model coefficients: X^2^ = 48.417, P = 0.000; Hosmer–Lemeshow goodness-of-fit test: X^2^ = 0.640, P = 0.726; −2 log likelihood = 38.522). In the linear regression analysis of these variables, VIF was <10. There were no problems of multicollinearity among these independent variables. The OR and 95% CI were measured through binary logistic regression*.

*NYHA, New York Heart Association; CPB, cardiopulmonary bypass; MAP, mean arterial pressure; ARDS, acute respiratory distress syndrome; CVA, acute cerebrovascular accident. Bold values represented P <0.05*.

## Discussion

This is the first study of independent risk factors for in-hospital mortality in patients with CRRT in a homogenous population of patients with AAAD.

In our study with 432 patients, the incidence of severe AKI (AKI stages 2–3), the overall in-hospital mortality, the proportion for requiring CRRT, and in-hospital mortality after CRRT, was 47.9, 15.3, 14.6, and 46.0%, respectively. Our findings revealed that diabetes, SCr, secondary thoracotomy, pulmonary infection, and severe AKI were the independent risk factors for CRRT. Preoperative severe heart failure, postoperative lactic acidosis, and a combination of any three or more risk factors were the independent risk factors for death in the patients with CRRT. The patients in the CRRT group had longer postoperative mechanical ventilation, higher hospitalization costs, and higher in-hospital mortality. This study supports evidence from the previous observations that 11–27% of patients undergoing surgery for aortic dissection required renal replacement therapy (RRT) (11–13) and the mortality for CRRT was between 40 and 70% (9, 14).

In this study, diabetes was found to be considered as an independent risk factor for CRRT that was consistent with the data published by The Japanese Society for Dialysis Therapy (JSDT), who found that diabetes has become the most common initial diagnosis for end-stage renal disease (ESRD) treated with dialysis (15). This is associated with hyperglycemia causing relative ischemia in the kidney, systemic endothelial cell dysfunction (16), podocyte injury (17), and sterile inflammation, leading to kidney injury and deterioration of kidney function (18).

Pulmonary infection was also an independent risk factor for CRRT. Sepsis (19), non-severe pneumonia (20), can give rise to the development and exacerbation of AKI and an increased risk of death due to the inflammatory response and organ crosstalk between lung and kidney (21).

Our findings were in accord with the recent studies indicating that the baseline SCr levels, second thoracotomy were considered as the independent risk factors for CRRT (11, 22, 23). Increased creatinine levels represent impaired kidney function (24). The second thoracotomy is performed when excessive bleeding occurs, which may cause hypovolemia (22). Perioperative hypotension or poor organ perfusion may lead to deterioration of renal function (25, 26).

Several reports have shown that serum cystatin C is a good indicator for identifying early renal impairment and predicting RRT requirements (27–30). Our study confirms that an elevated Cys-C is an independent risk factor for predicting the CRRT. Since serum cystatin C is not affected by diet, etiology of AKI, or urine output, it is a useful and highly diagnostic marker for AKI (27).

A severe and refractory AKI is an indication for CRRT, and early CRRT is recommended for patients with oliguria or fluid overload (31, 32). In our study, we found that AKI stage 2–3 was an independent risk factor for CRRT. CRRT can maintain water, electrolyte, acid-base balance, uremic solute homeostasis to prevent or delay the deterioration of renal function (32). The patients with AKI stage 2–3 had a 1-year survivability rate of 90% if the renal function was restored within 7 days (33, 34). It remains controversial as to the timing of initiation (35), dose, discontinuation (36), quality assessment indicators (37, 38) of RRT in patients with AKI. The clinicians must formulate and select individual treatment plans for their patients (39).

The results of the current study showed that in-hospital mortality in the CRRT group was 4.6 times higher than in the non-CRRT group. It was reported that close to 40% of the patients in the dialysis mortality population died from the causes, such as heart failure, stroke, and myocardial infarction and infection (15), drinking (40), CPB duration (41, 42), hypotension (43), CVA (44), hepatic failure, and ARDS (45). Our findings were coherent with those of the previous studies. The kidney may induce distant organ crosstalk, including lung, heart, liver, or intestine. The prognosis in complicated AKI is poor when distant organ injury occurs (46, 47). Therefore, to reduce dialysis mortality, it is important to treat the distal organ crosstalk caused by AKI rather than focusing on the renal impairment alone (48).

Previous studies had shown that renal malperfusion was an independent risk factor for AKI, 30-day mortality, and poor prognosis in patients with AAAD (5, 49, 50). In our study, there was no significant increase in the incidence of renal malperfusion and renal artery involvement before surgery in the CRRT group and the post-CRRT in-hospital mortality group relative to the other groups. It is probably because that renal malperfusion improved before surgery or because timely treatment after the occurrence of AKI prevented the progression of AKI and thus reduced the usage of CRRT and in-hospital mortality.

### Study Limitations

There were certain limitations to the present study: (i) data collected by different people over different periods may be affected by confounding factors, thereby affecting the conclusions; (ii) due to the lack of urine data, the creatinine level was used to define AKI; (iii) our study did not include long-term follow-up and did not clarify long-term outcomes, such as long-term mortality, whether the kidney function recovers after the CRRT or whether the condition progresses to chronic or end-stage renal disease after AAAD.

## Conclusions

In the patients with post-operative AAAD, CRRT was in high demand and in-hospital mortality remained high. Diabetes, baseline SCr, Cys-C, lung infection, second thoracotomy, and severe AKI were independent risk factors for CRRT. Severe cardiac failure and lactic acidosis were independent risk factors for in-hospital mortality in patients with CRRT. The risk factors for CRRT and in-hospital mortality in patients undergoing AAAD surgery were determined to help identify the high-risk patients and make appropriate clinical decisions. Further randomized-controlled studies are urgently needed to establish the risk factors for CRRT and in-hospital mortality.

## Data Availability Statement

The raw data supporting the conclusions of this article will be made available by the authors, without undue reservation.

## Ethics Statement

The studies involving human participants were reviewed and approved by Ethics Committees of the West China Hospital of Sichuan University. Written informed consent for participation was not required for this study in accordance with the national legislation and the institutional requirements.

## Author Contributions

LY, XC, LJ, and JZ: research idea and study design. XC, LL, and TZ: data acquisition. XC, MF, JY, XW, and SW: statistical analysis. LY, JZ, and LJ: supervision or mentorship. All authors contributed to the article and approved the submitted version.

## Funding

This study was supported by grants from the Sichuan Provincial Science and Technology Key R&D Projects [Nos. 2017SZ0113, 2017SZ0144, 2019YFS0282, and 2019YFG0491] and 1·3·5 Project for Disciplines of Excellence–Clinical Research Incubation Project, West China Hospital, Sichuan University [NO.2020HXFH049], Sichuan, China.

## Conflict of Interest

The authors declare that the research was conducted in the absence of any commercial or financial relationships that could be construed as a potential conflict of interest.

## Publisher's Note

All claims expressed in this article are solely those of the authors and do not necessarily represent those of their affiliated organizations, or those of the publisher, the editors and the reviewers. Any product that may be evaluated in this article, or claim that may be made by its manufacturer, is not guaranteed or endorsed by the publisher.

## Abbreviations

AKI: acute kidney injury
CSA-AKI: cardiac surgery-associated acute kidney injury
AAAD: acute type A aortic dissection
KDIGO: Kidney Disease: Improving Global Outcomes
AKI-D: AKI incidence requiring dialysis
eGFR: estimated glomerular filtration rate
SCr: serum creatinine
SD: standard deviations
IQR: interquartile range
BMI: body mass index
CKD: chronic kidney disease
COPD: chronic obstructive pulmonary disease
NYHA: New York Heart Association
AST: aspartate aminotransferase
ALT: alanine aminotransferase
DHCA: Deep hypothermic circulatory arrest
CPB: cardiopulmonary bypass
ALB: albumin
BUN: blood urea nitrogen
UA: uric acid
Cys-C: cystatin C
MAP: mean arterial pressure
AMI: acute myocardial infarction
CVA: acute cerebrovascular accident
ARDS: acute respiratory distress syndrome
CRRT: continuous renal replacement therapy.
